# Utilisation Patterns and Clinical Outcomes of Teleconsultation Versus Face‐to‐Face Follow‐Up of Breast Cancer Patients in the Post‐COVID‐19 Era

**DOI:** 10.1155/ijbc/6612877

**Published:** 2026-11-03

**Authors:** Hilda Wong, Timothy Robinson, Thomas Strawson-Smith, Amjed Alhilfi, Louise Panes, Thomas Wells

**Affiliations:** ^1^ Department of Oncology Bristol National Service Trust Bristol UK; ^2^ Bristol Medical School University of Bristol Bristol UK bristol.ac.uk

**Keywords:** breast cancer, cancer care, teleconsultation, telemedicine, teleoncology

## Abstract

**Background:**

There is a lack of updated data on the utilisation of teleconsultation after the end of the COVID‐19 pandemic. Objective clinical outcome measures to reflect its safety and effectiveness are underresearched.

**Methods:**

We retrospectively reviewed consecutive breast cancer outpatient clinic appointments which took place between 5 May 2023, and 30 June 2025, in the oncology department of a tertiary referral hospital. Patient characteristics, postconsultation acute services contact, emergency hospital admission rate and survival were compared between telephone and face‐to‐face consultation groups.

**Results:**

A total of 3098 consultations involving 433 patients were included in our analysis. The majority was face‐to‐face (66.1%). Teleconsultations were associated with a lower median age (*p* < 0.0001), higher proportion of patients with Eastern Cooperative Oncology Group performance status 0 (*p* = 0.0010), Stage III or IV disease (*p* = 0.0112), longer history of follow‐up (*p* < 0.0001) and active treatment (*p* < 0.0001). No significant difference was found in postconsultation acute services contact or emergency hospital admission rate after adjusting for confounding factors (*p* = 0.6165). Overall survival was similar in patients stratified by the proportion of teleconsultations out of all attended appointments (logrank *p* = 0.07), as was recurrence‐free survival (logrank *p* = 0.052) in early breast cancer.

**Conclusion:**

We reported ongoing high utilisation of teleconsultation in a real‐world breast oncology clinic setting and preliminary observation of its successful care delivery without compromise on safety and quality.

## 1. Introduction

Although the delivery of care virtually by telephone or video has long been practised in oncology [1, 2], it was the COVID‐19 pandemic that led to its renewed focus and widespread implementation, aiming to continue ambulatory care without the risk of exposure for patients and clinicians. Tertiary cancer centres across the world were reported to rapidly adopt remote care in up to 75% of outpatient visits early in the global outbreak, from a baseline of 1%–2% [3–8].

Beyond the acute phase of the pandemic, the proportion of virtual clinical encounters declined to a minority of 17%–20% in these institutions [3, 5, 6]. Given its potential to improve the availability and quality of healthcare delivery in favour of time and resources without distance restrictions [9], there is little debate that teleconsultation still has a role after the pandemic ended on 5 May 2023. However, updated data on its utilisation is scarce.

Extensive studies have shown high accessibility, feasibility, as well as patient and clinician satisfaction in telemedicine across general oncology clinics [10–12], various cancer types (such as colorectal [13], lung [14], breast [15, 16], head and neck [17], gynaecological [18] and urological cancers [19, 20]), patient subgroups (including palliative [21], older [22], low‐income [23] and rural [24] patients) and treatment modalities (chemotherapy [25] and radiotherapy [26]). On the other hand, concerns remain especially regarding communication of information and patient assessment and examination [5, 13, 19, 27]. More importantly, objective clinical outcomes are underresearched and there is a lack of solid evidence on the effectiveness and safety of teleconsultations [28–30].

Meta‐analyses which included very few cancer studies or not at all have reported no increase in mortality with telemedicine [31–34]. Clinical effectiveness as demonstrated in these reviews, in terms of biomedical measures such as blood pressure or blood sugar [31, 33], is not relevant in oncology. With regard to hospital admissions, contrasting findings ranging from 60% decrease to an increase of a similar percentage were reported [34]. Other studies suggested teleoncology could enhance patient outcomes, reduce complications and hospital admission [14, 35], but detailed data were not available.

Interestingly, breast cancer was rated in an article by oncologists as a top priority suitable for telehealth services [36]. As discussed, patient satisfaction and acceptance of teleconsultations in breast oncology are well‐established [15, 16, 23]. Nevertheless, apart from a study showing similar time to detection of recurrence in the telephone and hospital visit groups [37], little evidence on clinical outcomes exists.

The objectives of this work are therefore to provide updated information on the utilisation patterns of teleconsultations in breast cancer patients, compare real‐world clinical settings between remote versus in‐person consultations and investigate whether patient outcomes are affected by the means of delivery.

## 2. Methods

### 2.1. Study Site and Samples

We retrospectively reviewed consecutive breast cancer outpatient clinic appointments which took place between 5 May 2023, and 30 June 2025, in the oncology department of a tertiary referral hospital. The means of delivery of these consultations (face‐to‐face or telephone) were a shared decision between clinicians and patients. Whether they were new case or follow‐up appointments and routinely scheduled or urgent additional reviews were also recorded. All breast cancer patients, irrespective of stage, histology and treatment, were eligible. Their clinical condition at each consultation was retrieved from electronic medical records.

### 2.2. Clinical Outcomes

Data were analysed at either the consultation or patient level, depending on the nature of the outcome. Postconsultation Acute Oncology Service (AOS)/same‐day emergency care (SDEC) contact and emergency hospital admission (i) within 7 days and (ii) from Day 8 until 30 or next consultation, whichever was earlier, relate specifically to individual episodes of care and were evaluated at the consultation level. These outcomes were compared between the face‐to‐face and remote follow‐up groups. Elective admissions were excluded.

On the other hand, overall survival and recurrence‐free survival are inherently patient‐level outcomes. For these analyses, we stratified patients by the proportion of telephone out of all attended consultations into 0%, 1%–25%, 26%–50%, 51%–75% and 76%–100% to appropriately adjust for individuals who experienced a high volume of teleconsultations.

In the telephone cohort, whether an additional in‐person consultation was required to evaluate patient symptoms was noted.

### 2.3. Statistical Analyses

Analyses were performed in the R statistical environment, Version 4.5.1 (R Foundation for Statistical Computing). Descriptive statistics were used for patients′ characteristics. Median age was compared by Mann–Whitney test and categorical data by Pearson′s chi‐square test. Logistic regression was performed where appropriate. Survival was estimated using Kaplan–Meier method and compared by the log rank test. All tests were two‐sided, and a threshold of *p* < 0.05 was assumed for statistical significance.

### 2.4. Ethics Approval

The study is registered with the Department of Clinical Audit and Effectiveness at the University Hospitals Bristol and Weston NHS Trust, United Kingdom.

## 3. Results

### 3.1. Clinic Consultations

During the study period, there were 3165 breast cancer outpatient clinic appointments, in which 67 were unattended and therefore excluded. A total of 3098 consultations involving 433 patients were included in our analysis (Figure 1). The majority of these clinical encounters were face‐to‐face (66.1%, *n* = 2053).

**Figure 1:**
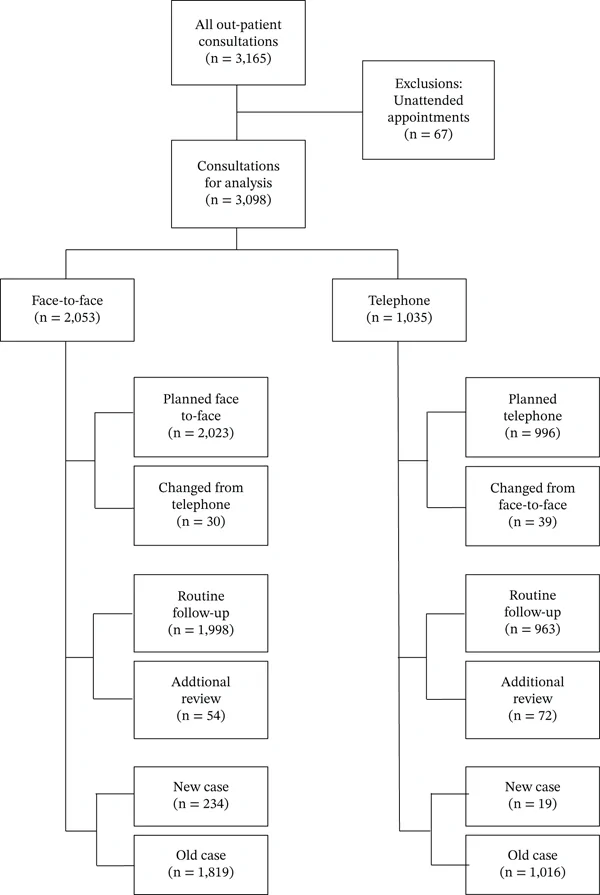
Telephone and face‐to‐face outpatient clinic consultations during the study period.

The means of delivery was changed at the consultation in a small percentage (1% from telephone to face‐to‐face and 4% vice versa). Reasons for changing to a face‐to‐face visit included comprehensive review after recent hospitalisation (17%, *n* = 5), patient attending hospital for another reason (13%, *n* = 4), physical examination requested by clinician (3%, *n* = 1) and patient preference (7%, *n* = 2). As for changing to a telephone consultation, most did so because of physical illness (46%, *n* = 18), logistic reasons (26%, *n* = 10) or patient preference (13%, *n* = 5). Telephone consultations were more often used for urgent additional reviews (*p* < 0.0001), whereas new case appointments were more frequently face‐to‐face (*p* < 0.0001).

### 3.2. Means of Delivery According to Date

From May to December 2023, 64% of clinic appointments were conducted face‐to‐face (Figure 2). The proportion of teleconsultations decreased from 36% to 32.2% in the first half of 2024 but then slightly increased to 32.6% in the latter half of the year and to 32.9% in the remainder of our study period in 2025.

**Figure 2:**
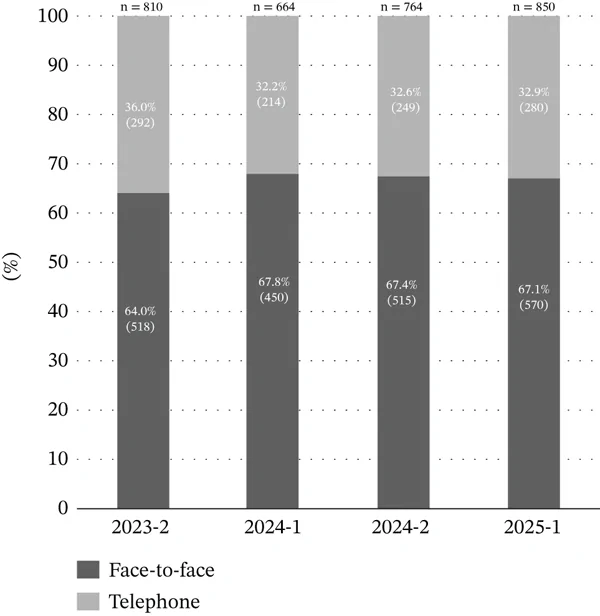
Trend of teleconsultation uptake according to date.

### 3.3. Patient Characteristics

Among the 433 patients in our study, all except three (0.7%) were female (Table 1). The age distribution was similar to local epidemiology [38]. Our cohort was enriched with Stages III and IV cancers as compared with latest data [39], reflecting the caseload in a tertiary referral oncology centre. During the study period, the patients had a median of 4 but up to 45 clinic consultations. Approximately one‐third had face‐to‐face consultations only, whereas most had a combination of remote and in‐person appointments. Median follow‐up time was 16.2 months (range 0.0–282.0 months).

**Table 1:** Characteristics of patients in the study.

Patient characteristics	% (no. of patients)
Sex	
Female	99.7 (430)
Male	0.7 (3)
Age	
Median (range)	63 (31–92)
≤ 35	1.2 (5)
36–50	15.0 (65)
51–80	72.5 (314)
> 80	11.3 (49)
Disease stage at last follow‐up	
0	2.8 (12)
I	19.9 (86)
II	35.6 (154)
III	18.0 (78)
IV	22.9 (99)
Median no. of consultations (range)	4 (1–45)
Proportion of teleconsultations (%)	
0	34.6 (150)
1–25	18.2 (79)
26–50	29.6 (128)
51–75	10.4 (45)
76–100	7.2 (31)
Median follow‐up (range) (months)	16.2 (0.0–282.0)

Total number	433

### 3.4. Clinical Conditions at Consultations

Teleconsultations, as opposed to face‐to‐face encounters, were associated with a lower median age (57 vs. 60, *p* < 0.0001) and notably a lower percentage of patients aged over 80 (4.1% vs. 8.0%, *p* < 0.0001) (Table 2). A higher proportion of patients with Eastern Cooperative Oncology Group (ECOG) performance status (PS) of 0 were recorded in the telephone group, with more patients with PS 2 or 3 in the face‐to‐face group (*p* = 0.0010). In contrast, there was a slightly greater percentage of patients with Stage III (22.9% vs. 18.2%) or IV disease (31.7% vs. 30.6%) in the telephone group (*p* = 0.0112). Patients who had been on follow‐up for longer tended to have telephone consultations (*p* < 0.0001).

**Table 2:** Clinical conditions at telephone versus face‐to‐face consultations.

Clinical conditions at consultation	Face‐to‐face [% (no. of consultations)]	Telephone [% (no. of consultations)]	*p*
Age			
Median (range)	60 (29–92)	57 (30–91)	< 0.0001
≤ 35	2.8 (58)	3.4 (35)	
36–50	20.2 (415)	24.7 (256)	
51–80	69.0 (1416)	67.8 (702)	
> 80	8.0 (164)	4.1 (42)	< 0.0001
ECOG PS			
0	48.4 (994)	57.2 (592)	
1	18.5 (379)	17.2 (178)	
2	3.0 (62)	1.4 (14)	
3	1.2 (25)	0.9 (9)	0.0010
Disease stage			
0	1.4 (28)	1.3 (13)	
I	12.3 (253)	11.0 (114)	
II	37.3 (765)	32.8 (339)	
III	18.2 (373)	22.9 (237)	
IV	30.6 (629)	31.7 (328)	0.0112
Time on follow‐up			
≤ 3 months	1.2 (25)	1.0 (10)	
3.1–12 months	29.6 (608)	16.4 (170)	
12.1–24 months	37.5 (770)	35.0 (363)	
24.1–60 months	23.7 (487)	29.9 (309)	
> 60 months	7.9 (163)	17.7 (183)	< 0.0001
Undergoing treatment	70.9 (1456)	84.2 (871)	< 0.0001
*Treatment intent*			
*Adjuvant*	*55.1 (802)*	*60.7 (529)*	
*Neoadjuvant*	*10.3 (150)*	*6.7 (58)*	
*Palliative*	*34.6 (504)*	*32.6 (284)*	0.0036
*Treatment type*			
*Chemotherapy and/or targeted therapy and/or immunotherapy*	*37.0 (538)*	*45.7 (398)*	
*Endocrine therapy with or without targeted therapy*	*50.3 (732)*	*51.2 (446)*	
*Radiotherapy*	*10.9 (159)*	*1.1 (10)*	
*Others*	*1.9 (27)*	*2.0 (17)*	< 0.0001
*Toxicities*			
*Grade 3 or above*	*5.1 (74)*	*3.7 (32)*	0.1125
*Recurrent grade 2*	*7.8 (114)*	*7.0 (61)*	0.4563
*Response (for advanced disease where assessment is due)*			
*Partial or complete response*	*26.9 (169)*	*27.1 (89)*	
*Stable disease*	*26.9 (169)*	*31.1(102)*	
*Progressive disease*	*10.8 (68)*	*7.9 (26)*	
*Not due for assessment*	*35.5 (223)*	*33.8 (111)*	0.2140
*Treatment decision*			
*Dose reduction*	*5.6 (81)*	*6.0 (52)*	0.6911
*Treatment change*	*10.4 (151)*	*6.3 (55)*	0.0008
Additional visit needed	0 (0)	0.8 (8)	< 0.0001

Total number	2053	1035	

*Note:* There were no missing data in any of the variables, except the following: ECOG, 28.9% (*n* = 593) in face‐to‐face and 23.3% (*n* = 242) in teleconsultation cohorts, and stage, 0.2% (*n* = 5) and 0.3% (*n* = 4), respectively. Missing data were handled by retaining them as a distinct ‘missing’ category, ensuring that the denominator for each variable remains the total cohort size (*n*). Unless otherwise stated, denominators correspond to the total number of consultations (*n* = 2053 for face‐to‐face and *n* = 1035 for teleconsultation cohorts). The exceptions (italics area) are as follows: Number of consultations involving active treatment was used as the denominator for treatment intent, type, toxicities and treatment decision (*n* = 1456 and *n* = 871, respectively); number of consultations involving active treatment in advanced setting was used as denominator for response (*n* = 629 and *n* = 328, respectively). Occurrence of toxicities of Grade 3 or above and recurrent Grade 2 were not mutually exclusive.

The majority of consultations in both groups were conducted for patients undergoing active cancer treatment, although a higher proportion was observed in the telephone group (84.2% vs. 70.8%, *p* < 0.0001). Of these treatments, 60.7% in the telephone group were adjuvant intent (vs. 55.2% in the face‐to‐face group), whereas only 6.7% were neoadjuvant (vs. 10.3%, *p* < 0.0001). Chemotherapy, targeted and/or immunotherapy were discussed in 45.7% of telephone and 37.0% of face‐to‐face consultations, compared with radiotherapy in 1.1% and 10.9%, respectively (*p* < 0.0001). There was no significant difference in the distribution of patients having moderate to severe toxicities or attending for scan results (including those showing progressive disease) in the two groups of consultations. Both managed a similar proportion of dose reductions, but treatment changes took place in a larger percentage of face‐to‐face consultations (10.4% vs. 6.3%, *p* < 0.0001).

Finally, an additional assessment for physical examination was required after a small number (8 of 1035) of telephone consultations.

### 3.5. Clinical Outcomes

All‐cause and cancer‐specific emergency service use at various time points are summarised in Table 3. The rate of AOS/SDEC contact was not shown to be higher with teleconsultations. Although a larger percentage of remote follow‐up appointments led to emergency hospital admission within 7 days, no statistically significant difference was found after adjusting for confounding factors by logistic regression (adjusted odds ratio 1.82, 95% CI 0.93–3.58, *p* = 0.080) (Table 4). Instead, Stage IV disease was an independent predictor of early emergency hospital admissions, more than doubling the odds compared with Stage II (adjusted OR 2.35, 95% CI 1.08–5.42, *p* = 0.036). Nonchemo, ‐targeted or ‐immunotherapy treatments such as hormone and bone‐directed agents, potentially reflecting lower risk disease or stable disease control, showed a marginal trend towards lower admission odds (adjusted OR 0.34, 95% CI 0.11–1.05, *p* = 0.058).

**Table 3:** Postconsultation acute medical services contact and emergency hospital admissions in telephone versus face‐to‐face groups.

Emergency services contact	Face‐to‐face [% (no. of consultations)]	Telephone [% (no. of consultations)]	*p*
All‐cause emergency services contact			
AOS/SDEC contact within 7 days	2.6 (54)	2.3 (24)	
AOS/SDEC contact on Day 8 until 30 or next consultation	6.2 (127)	4.2 (43)	
Hospital admission within 7 days	0.9 (18)	1.8 (19)	
Hospital admission on Day 8 until 30 or next consultation	3.3 (68)	2.4 (25)	0.0160
Cancer‐specific emergency services contact			
AOS/SDEC contact within 7 days	2.3 (47)	2.0 (21)	
AOS/SDEC contact on Day 8 until 30 or next consultation	5.6 (114)	3.4 (35)	
Hospital admission within 7 days	0.9 (18)	1.7 (18)	
Hospital admission on Day 8 until 30 or next consultation	2.8 (57)	2.2 (23)	0.0186

Total number	2053	1035	

**Table 4:** Multivariate logistic regression analysis of factors affecting 7‐day emergency hospital admission. Stage I patients were excluded from the multivariable model because of zero admission events (complete separation).

Variable	Adjusted odds ratio	95% confidence interval	*p*
Means of delivery			
Face‐to‐face	1.00	Reference	—
Telephone	1.82	0.93–3.58	0.080
Disease stage			
II	1.00	Reference	—
III	1.35	0.48‐3.55	0.549
IV	2.35	1.08–5.42	0.036
Undergoing treatment			
Not on any treatment	1.00	Reference	—
Chemotherapy, targeted therapy and/or immunotherapy	1.85	0.81–4.79	0.168
Other treatment (e.g., hormonal or bone‐directed agents)	0.34	0.11–1.05	0.058
Age (per year)	1.00	0.97–1.03	0.974

Total number	2053	1035	

Survival data was immature, with only 42 deaths and 17 relapses recorded during the study period. Preliminarily, overall survival was not significantly different in patients who had various proportions of teleconsultations (logrank *p* = 0.07, Figure 3A). In Stage IV patients specifically, similar findings were shown (logrank *p* = 0.092, Figure 3B). Furthermore, there was no difference in disease recurrence rate (*p* = 0.2194) or recurrence‐free survival (logrank *p* = 0.052) in early breast cancer patients stratified by the proportion of teleconsultations.

**Figure 3:**
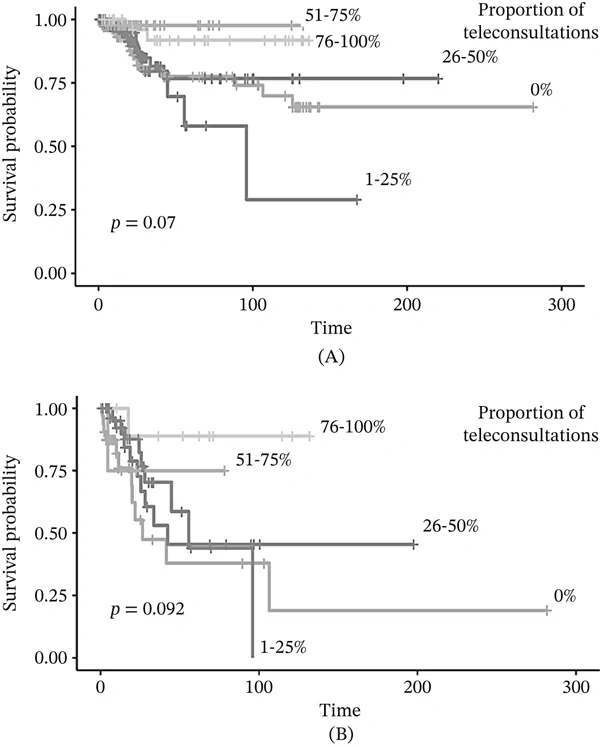
Preliminary overall survival of patients as stratified by their proportion of teleconsultations out of all attended appointments. (A) All patients. (B) Stage IV patients.

## 4. Discussion

Our survey showed that, 2 years on from the end of the pandemic, teleconsultations were still conducted in nearly one‐third of breast cancer outpatient appointments, compared with 17%–20% reported in the literature [3, 4, 6]. One of the factors for the relatively low utilisation rate in these institutions was billing and reimbursement issues, whereas in the United Kingdom, most healthcare services are free at the point of use. On the other hand, in centres where teleconsultations are more often used, physical distance from medical facilities and lack of primary healthcare providers are considered the main reasons [40]; these are also less applicable in our setting. Our high uptake rate of teleconsultation more likely reflects ready acceptance by patients and clinicians. Indeed, its utilisation appeared to be stable or even increasing during our study period. As such, research evidence to confirm noninferiority in its safety and clinical outcomes has never been more important to support its ongoing use.

This was timely assessed in the current study. We recognise that cancer and its treatment are complex and acute complications often occur; thus, we reported novel outcome measures, such as 7‐ or 30‐day acute oncology contact or hospital admission rate after consultations, to provide a quantitative account of clinical effectiveness and quality of care delivered at the appointments. Our findings showed that only small and similar percentages of patients required acute services and/or emergency admissions after telephone versus face‐to‐face consultations. Preliminary survival data were also reassuringly comparable.

We noted a trend, albeit statistically insignificant, for better overall survival in patients who had a higher proportion of their consultations in telephone format. This was still the case when controlled for disease stage and only Stage IV patients were analysed. This might reflect the selection of more clinically stable or lower risk patients for teleconsultations but not necessarily those with early stage disease; there was in fact a higher percentage of Stage III/IV patients in the telephone group.

Given that our telephone cohort achieved similar outcomes as the standard, it might be reasonable to infer that they represented the patients suitable for this mode of consultation, as summarised in Table 5 as potential indicators for patient selection. These include younger patients (up to 50–60 years of age), good ECOG PS 0 or 1 and ideally on follow‐up for 12–24 months or longer, regardless of stage, treatment toxicity or need for dosage reduction. On the other hand, patients on neoadjuvant treatment, new patients and those who have progressive disease and/or require a treatment change are underrepresented in our telephone cohort thus better seen in person. The latter is consistent with the three eligibility criteria identified by other researchers, namely, ‘the consultation concerned should not be the first consultation, the patient should be a known patient that the doctor trusts not to minimise the description of symptoms and the results of the patient′s evaluations and examinations must be good’. [41] Patients who do not meet our criteria may still benefit from teleconsultations, although outcome measures are less clear from our data and clinical discretion is to be exercised.

**Table 5:** Potential indicators for patient selection for teleconsultations.

Patient characteristics	Potential indicators for selection
Age	Up to 50–60 years
ECOG PS	0–1
Time on follow‐up	Over 12–24 months
Type of appointment	Old case
Disease stage	Any
Treatment intent	Adjuvant or palliative
Treatment toxicities	Any
Dose reduction	Any
Treatment change	None
Treatment response	Stable disease or better

A few patients in the telephone group needed an additional in‐person appointment, suggesting the continued role of physical examinations in clinical encounters. By the same token, patients on neoadjuvant treatment or radiotherapy, requiring close monitoring of clinical response and skin toxicities, respectively, were more often seen in the face‐to‐face group. It also highlights that awareness of this pitfall and a low threshold for supplementary assessment are important, although the minimal rate of these assessments in our study does not preclude teleconsultations from cancer care.

The current study was limited by the modest patient number and relatively short follow‐up. The interpretation of comparable clinical outcomes warrants caution; although multivariable logistic regression adjusted for measurable clinical differences between cohorts, residual selection bias prevents attributing outcomes solely to consultation modality. There were also several aspects in telemedicine that we did not address, such as the timeliness of medication dispensing and delivery after teleconsultations or telepharmacy [42], equitability, efficiency and ethics. Patient or clinician perception and satisfaction were not within the scope of this research either, although this perspective has been well reported in the literature [15, 16]. Only telephone, but not other digital tools including videoconferencing or short‐text messaging, were evaluated in this analysis. Finally, we included specifically breast cancer patients, so our results might not be generalisable.

In conclusion, we reported ongoing high utilisation of teleconsultation in a real‐world breast oncology clinic setting and preliminary observation of its successful care delivery without compromise on safety and quality, as reflected by clinical results. The granularity of data in our study provided insights on patient characteristics to inform selection and on outcome measures that are otherwise difficult to quantify. Our findings contributed towards evidence‐driven and patient‐centred development of contemporary cancer care models.

## Funding

No funding was received for this manuscript.

## Ethics Statement

The study is registered with the Department of Clinical Audit and Effectiveness, University Hospitals Bristol and Weston NHS Trust, United Kingdom, on 1 July 2025. The need for ethics approval and patient consent for the collection, analysis and publication of the retrospectively obtained and anonymised data for this noninterventional study was waived.

## Conflicts of Interest

The authors declare no conflicts of interest.

## Data Availability

The data that support the findings of this study are available from the corresponding author upon reasonable request.
